# microRNA-145 inhibits osteosarcoma cell proliferation and invasion by targeting ROCK1

**DOI:** 10.3892/mmr.2022.12790

**Published:** 2022-07-04

**Authors:** Pengfei Lei, Jie Xie, Long Wang, Xucheng Yang, Zixun Dai, Yihe Hu

Mol Med Rep 10: 155–160, 2014; DOI: 10.3892/mmr.2014.2195

Following the publication of this paper, it was drawn to the Editors’ attention by a concerned reader that several of the data panels shown in Fig. 4A were overlapping with panels contained within the same figure, where the panels were intended to portray the results from a range of different cell migration assay experiments. Given the number of overlaps of data that have been identified, the Editor of *Molecular Medicine Reports* has decided that this paper should be retracted from the Journal on account of a lack of confidence in the presented data. The authors were asked for an explanation to account for these concerns, but the Editorial Office did not receive a satisfactory reply. The Editor apologizes to the readership for any inconvenience caused.

